# How We Built Workplace Based Assessment-for-Learning in Irish GP Training

**DOI:** 10.5334/pme.1428

**Published:** 2025-07-22

**Authors:** Karena Hanley, Edward McSwiney, Brian O. Malley, Aileen Barrett, Brian McEllistrem

**Affiliations:** 1 GP Training Unit, Irish College of General Practitioners, Dublin, Ireland

## Abstract

**Introduction:**

Programmatic Assessment displays the comprehensive picture of a learner’s competence through selection of assessment methods and design of organisational systems [1]. This paper describes how the Irish College of GPs (ICGP) designed and implemented a new, national, workplace-based assessment (WBA) system for GP training as part of an ongoing evolution towards Programmatic Assessment, with a focus on assessment-for-learning [1].

**Methods:**

Six overlapping workstreams over five years led to success: iterative consultation and design, entrustable professional activities, software design, stepwise implementation, separation of mentor/assessor roles and WBA training embedded in feedback literacy and growth mindset learning.

**Results:**

Our design focused on collecting longitudinal, low stakes assessments organised into core competences in a manner to support learners. 18 entrustable professional activities were developed and implemented, along with a software platform designed to enter and display accumulated data. Competence committees assess both qualitative and quantitative data periodically on the learner’s journey to oversee progression and make high stakes decisions. We describe the development of the system along with aids and barriers to its adoption.

**Discussion:**

Structured continuous consultation with the training community and constant reference to the educational literature were both important for success. Novel features of our system are the distancing of mentor and assessor roles, the avoidance of recommended minimum numbers of WBA entries, and consideration of the validity and reliability of the system as a whole rather than of the tools.

## Background and Need for Innovation

The Irish College of GPs, responsible for postgraduate general practice (GP) specialist training in Ireland, underwent transformational change between 2018 and 2023. Prior to 2021, the Health Service Executive (HSE) had responsibility for the fourteen independent, regionally developed training schemes which delivered GP vocational training. The Irish College of GPs was responsible for the summative membership examinations and accreditation of the schemes. In 2021, responsibility for GP postgraduate training was transferred from the HSE to the Irish College of GPs, along with amalgamation of two schemes, leaving thirteen schemes in total. This training transfer occurred at a time of rapid expansion in the number of commencing doctors – the new trainee intake to GP training has increased from 170 new trainees in July 2017 to 350 in July 2024.

A review of assessment structures set the Irish College of GPs on a course towards programmatic assessment (PA) [2,3]. Prior to the GP training transfer, trainees submitted paper-based logbooks as evidence of their workplace learning and performance. WBA was wholly reliant on in-training evaluation reports (ITERs), with significant variation in how trainees were assessed across the fourteen schemes. A nationally implemented system of WBA, along with a redesign of the portfolio review was needed.

PA proffers a model of longitudinal, low-stakes assessment, with multiple assessment methods and tools, each aligned to a core competence valued by the discipline or profession [4]. Developing competence relies on, among other things, a mutually respectful and trust-based trainee-supervisor relationship [5] for which evidence has supported the use of ‘entrustable professional activities’ (EPAs) [6,7,8]. Facilitation of narrative feedback and a mix of qualitative and quantitative elements in formative assessments have been shown to assist learning strategies [9]. These became our guiding concepts from the outset. Lessons from previous PA implementations [10] suggest that translating evidence into practice must adapt to the context, culture and previous structure of the programme. It requires committed informed leadership, and agile responses to the complex systems. We differ from previous implementations in that we did not concurrently redesign our curriculum.

## Goal of Innovation

The goal of our innovation is to introduce an acceptable new national WBA system which promotes learning, aligning with international directions in competency based medical education. This meant creating a robust, longitudinal, continuous low stakes assessment system which tailors learning to an individual trainee’s needs, and provides multiple scaffolded micro judgements from multiple assessors [1,11].

A review of the literature identified pitfalls to avoid, which include WBAs being viewed as tick box exercises [12], leaving WBAs until late in the rotation [13,14], completing WBAs long after the ‘event’ [14], and having the minimum recommended number of observations becoming the maximum number actually completed [13].

We noted concerns about the validity of individual observations within WBAs [14,15], and lessons about faculty and trainee resistance to using WBAs [16] especially if found to be too time consuming [5]. We embraced recent advances in technology-enabled learning, avoiding paper entirely. The rationale behind our starting point was published previously [17], where we explored the question “What do GP trainers, trainees and Programme Directors understand as the purpose and function of WBA?”. While concerned about the work implications of WBAs, respondents were positive about using WBAs to structure quality feedback. They incorrectly viewed WBAs as high stakes, with trainers disliking heavy assessment responsibilities. This prompted early adoption of the terms “feedback-for-learning” and “assessment-for-learning”.

## Steps Taken for Development and Implementation

### 1. Iterative design and consultation

**Figure 1:**
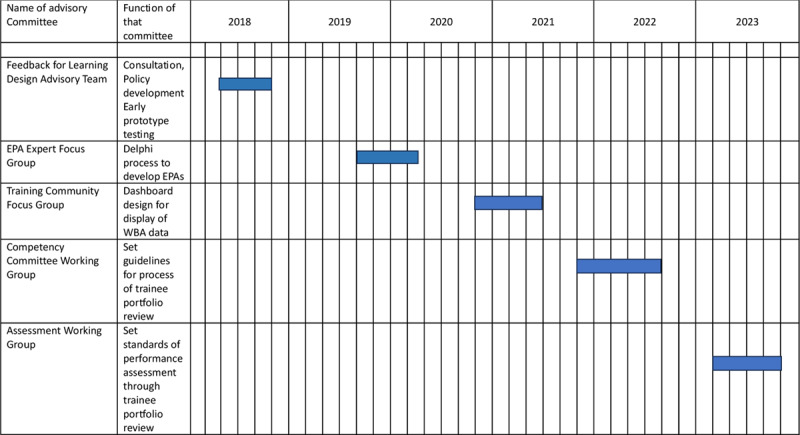
Advisory committees, their function and the periods over which they met. Each of these committees had two to four representatives from each key stakeholder group including GP trainees, clinical supervisors (GP trainers), college faculty, and external expert educational advisors. College faculty included programme directors, the Chair of Examiners and the National Lead on Assessment. The latter two were at every advisory committee, with different individuals representing their groups otherwise. The advisory committees held five to eight meetings each.

Insufficient consultation with the training community hinders the development of acceptable assessment structures [18]. We continuously sought out the views of clinical trainers, faculty, and other key stakeholders. Change management was achieved by a repeated cycle of design followed by broad consultation, adaption, and then redesign. Five sequential advisory committees, shown in Figure 1, were answerable to the college governance committee for GP training, ensuring ongoing stakeholder representation in the oversight of assessment development. Updates were presented to every national faculty, GP trainer and trainee conference from 2018 onwards. We also collaborated with hospital teachers and provided regular reports to the National Doctor’s Training and Planning Unit (NDTP) of the Health Service Executive (HSE) of Ireland and to the Irish Medical Council.

### 2. Entrustable Professional Activities

**Figure 2:**
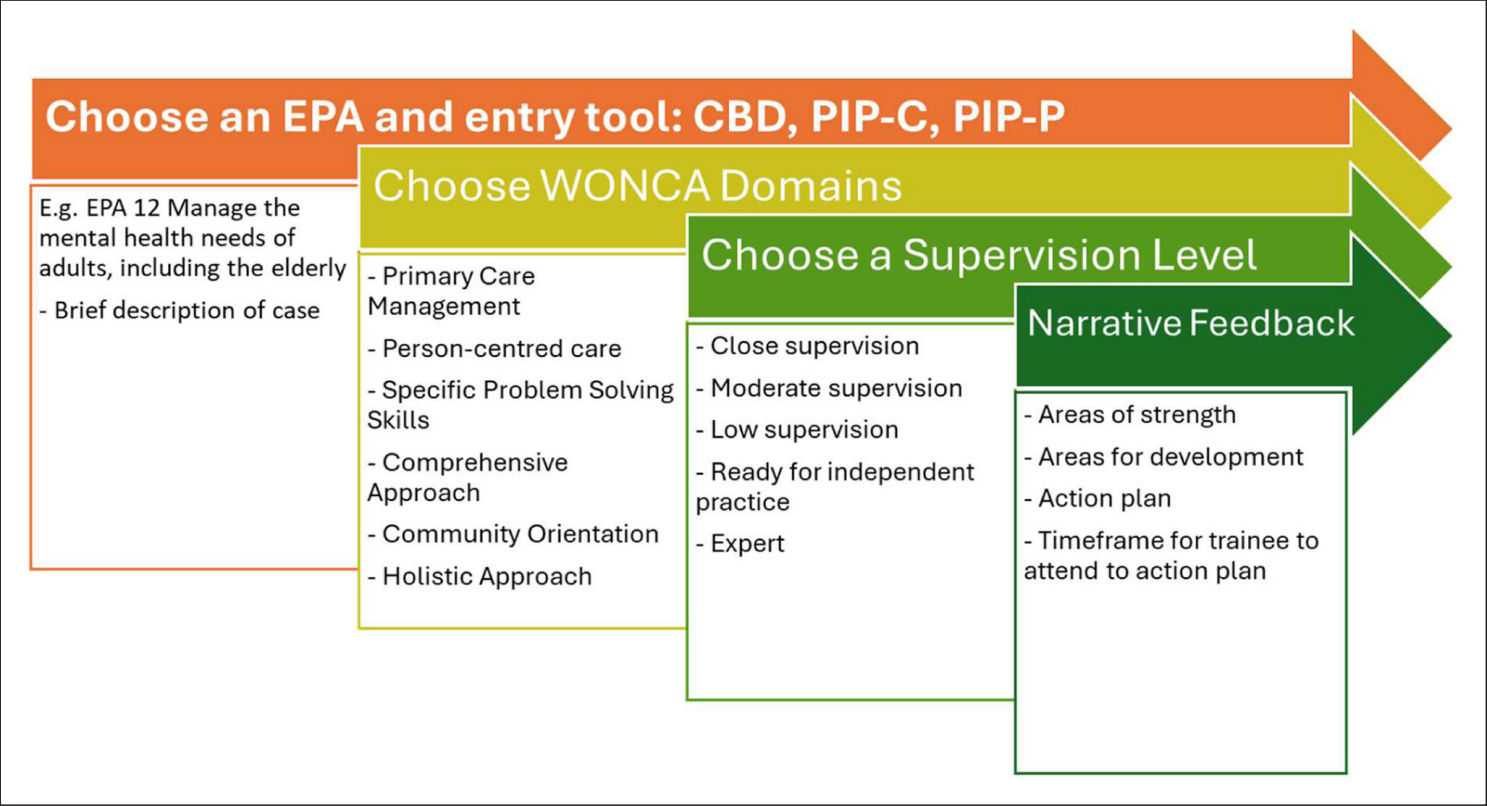
Entering a learning record under an EPA on the software platform.

Bespoke WBAs had been developed for Irish GP training in 2017 [19]. However, on piloting it became evident that these learning tools, (the Performance in Practice-Consultation (PIP-C) and Performance in Practice-Procedure (PIP-P)), needed to be more clearly aligned to a curriculum framework to be of value to trainees and trainers. We therefore embarked on the development of an EPA framework. A modified Delphi process, conducted in 2019, created a set of 18 EPAs, available as supplementary file 1. Two full-day workshops were conducted with 19 stakeholder representatives, similar in make-up to the advisory committees, but with two educational advisors. In the first workshop (November 6^th^ 2019), small groups created a list of 20 EPAs suitable for Irish General Practice. This work was guided by sample EPAs for General Practice from Canada [7] and the EQUAL quality criteria [20]. The workshop was followed by a three round Delphi in November/December among 40 GPs. The GPs were asked to rate the initial list answering the question ‘What are the important and essential tasks of the GP?’, and to propose any other EPAs. The criterion for inclusion of an EPA cut-was >80% agreement, No new EPAs were included and two were dropped. The second workshop (January 8^th^, 2020) with the same 19 representatives confirmed the final choice of EPAs and created the unique descriptors of competent practice for each EPA. Sub-competencies are mapped to the six domains (also called core competencies) of the World Organisation of Family Doctors (WONCA) European Definition of General Practice [21], thereby aligning with our curriculum [19]. Benchmarking was achieved by using descriptors, adapted from the literature [22,23] for expected supervision levels for each domain in each EPA. The PIP-C and PIP-P are combined with a generic case-based discussion (CBD) tool, giving trainees a choice of three WBA tools with which to enter a learning event in each EPA (Figure 2).

### 3. Software Design

We examined 17 software providers before choosing and co-developing a bespoke WBA software platform [24], we named “ICGP EPA”. Trainees are encouraged to seek regular feedback and to document that feedback, including an entrustment judgement, (supervision level) in one or two EPA domains. Each entry, shown in Figure 2, is a brief record of a verbal feedback conversation. Guided by the descriptors of competent practice for each EPA, and the descriptors of supervision levels for each of the domains under each EPA, trainees are encouraged to construct their own learning paths towards readiness for independent practice [25], passing through their normal educational and clinical activities. Feedback can come from multiple service providers, e.g. nurses, physiotherapists, administrative staff, so long as the feedback giver has superior knowledge on the item in question. The oversight of that feedback remains with the trainee’s clinical and educational supervisors. By accumulating multiple entries over time, a picture of growing ability builds, based on entrustment judgements and narrative feedback, depicted on the trainee EPA dashboard. This data, along with data from other sources, enables the competency committees to make high stakes progression decisions.

**Figure 3:**
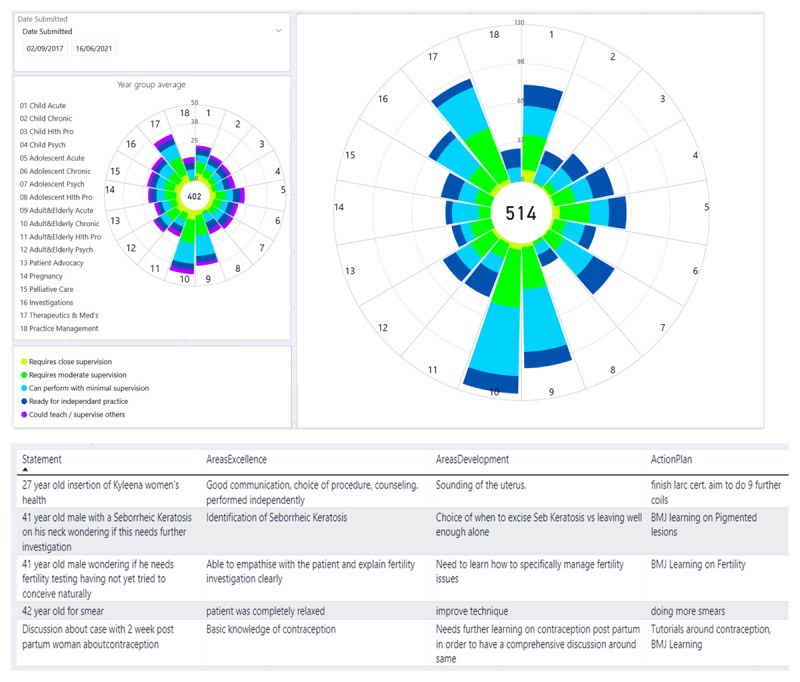
The trainee dashboard of accumulated WBA entries per EPA. Example of accumulated supervision levels of a (fictional) trainee over 1 year. The length of each spoke related to the number of observations in that EPA, with colour coding of the supervision level judgements; yellow – needs high supervision, green – needs moderate supervision, light blue – needs low supervision, dark blue – ready for independent practice, purple – exceptional. Underneath the graph is an example of the recorded narrative feedback from EPA 11. The ability to drill down into the narrative feedback exists for each EPA.

In the depiction of data from trainee WBAs, the summary of their activity level and entrustment progression, the distribution of activity and progress over EPAs and domains, the ability to drill down into cases and feedback with ease and the differing views and access for trainees, supervisors and faculty all need careful consideration. This we achieved in a process already published [26]. Our dashboard of summary data, used in the first years of piloting and implementation, as shown in Figure 3, displays information which can be understood at a glance. The pattern demonstrated in Figure 3 is consistent with the final year of training, approaching readiness for independent practice. Every EPA has received attention from this trainee, with EPA 9 and 10 having the greatest number of entries. Distribution of the supervision levels can be seen for the training year to date, with the option of customising the date range to view any specified period. Filters can be applied by supervision level, WBA tool use (PIP-C, CBD, PIP-P) and by custom date range. Furthermore, the EPA dashboard enables the ability to drill down to the narrative feedback on individual entries. The EPA dashboard updates in real-time, the same view is available simultaneously to the trainee, their clinical supervisor and their educational supervisor(s). Our data entry and data display screenshots are available in Supplementary file 3.

The EPA dashboard is also utilised by the training scheme competency committees. They use dashboard pattern recognition and sampling as part of the assessment of trainee progress during high stakes competency progression assessments, as shown in supplementary file 2. The competency progression committees also take into account the whole portfolio of trainee accomplishment: clinical placement reports, examination results, day release engagement, and virtual learning engagement.

### 4. Stepwise Implementation

Implementation of EPAs has been achieved in a consultative, iterative fashion similar to their design. From 2019, we conducted assessment workshops with trainers, trainees and faculty staff, averaging ten to twelve regional workshops each year. This continued throughout the Covid pandemic when workshops became virtual. We ran workshops at every annual National Trainer’s Conference, National Programme Director’s Conference and most National Trainee Conferences. We created a dedicated area on the ICGP website on which we placed explanatory video recordings and documents. We welcomed and responded to email queries continually. Multiple encounters with stakeholders allowed necessary ventilation of fears and concerns, coupled with EPA tailoring and software development to ensure best fit. Four pilots were undertaken with increasingly large cohorts, starting with the new trainees who commenced training in 2021. In July 2023, WBA using EPAs was made mandatory for all trainees, except those who commenced GP training before 2021.

Competition between clinical demand and educational responsibilities, in particular assessment documentation [5], is a barrier to effective clinical supervision [27]. Our tools can be completed quickly, with the goal that feedback is attained in as close a time to the learning interaction as possible. When discussing EPAs with the training community, EPA records are likened to patient records in their importance. Absence of wi-fi at some clinical sites made it necessary to record EPA WBA entries using a smart phone or laptop with later automatic synchronisation.

It is the trainee’s responsibility to record sufficient entries to show a clear pattern of acquisition of competence. This means showing progressively higher entrustment judgements over time, and a pattern of feedback across EPAs and EPA domains consistent with a normal training trajectory. That pattern takes continued application and continued recording, with variation expected from trainee to trainee and according to their clinical placements. We did not set a minimum number of entries, which would have created an arbitrary target. This approach also avoids a culture where more enthusiastic trainees could feel inhibited from creating entries for their own benefit as a stated minimum number of entries becomes the expected number by all. Volume of recorded data remains a driving force as sufficient records are the first criterion for the competency committee in making high stakes decisions. The data, or just as valuable, lack of data, are material for conversations with trainees who are falling off the normal training trajectory.

### 5. Separation of the mentor and assessor roles

Fear of the assessor role interfering with the teacher role [5] was evident in our training community. Along with their immediate clinical supervisor (the Trainer) who is their principal mentor, each GP trainee is assigned their own educational supervisor, who is offsite, and who also monitors their dashboard. Educational supervisors are mainly GPs who are also part-time college employees. Educational supervisors staff the day release theoretical course which all trainees attend weekly during the academic year. The educational supervisors oversee the progress of their cohort of trainees through frequent oversight of their recorded data and twice annual one to one supportive review. They also form the bulk of scheme competency progression committees described above. The competency committee has clinical supervisor representatives who do not participate in decisions on their own trainee. It is this committee that makes all the high stakes decisions in conjunction with the training body. Meaningful triangulation of data is facilitated by a mixture of qualitative (narrative) and quantitative data [28,29,30] which is discussed by competency committees, producing diagnostic feedback to trainees. Evidence based policies and procedures [31,32,33,34,35] have been developed for the competency committees to manage every type of training trajectory, including a failing trajectory.

Nonetheless, formative assessments are often perceived by learners as being mini summative assessments [2,15]. Our focus on the critical value of clinical supervisors in providing longitudinal low stakes formative assessment, with the competency committee making the high stakes judgements, weakens this perception. We also distance the single observation from inference of overall competence in that area, preceding every entry with the statement ‘*…competence/supervision levels can vary considerably, therefore the judgements in domains relate to a given case at a given point in time and are not an overall judgement of a trainee’s competence in the area*’. This emphasises each entry as low stakes, a pixel contributing to the overall picture.

### 6. WBA training embedded in training in feedback literacy and growth mindset learning

The relationship between teacher and learner should be an educational alliance [5]. Feedback literacy and a mature self-directing attitude to their learning by trainees enhances that educational alliance. Training in the software has been accompanied by training in feedback literacy and growth mindset learning. We have endeavoured to flatten the hierarchy between teacher and learner, with each considering the other’s perspective [36]. The emotional impact of feedback must be acknowledged, as this ameliorates its negative effects [37]. Learners must take responsibility for agreed actions which result from feedback. Learners who have availed of feedback literacy training describe this as an ‘awakening’ [38], with particular value placed on workplace-based feedback.

Teaching about growth mindset learning fosters a willingness to record entries where high supervision levels are needed [39], as would be expected in the earlier years of training. In contrast to fixed mindset learners, growth mindset learners do not hide their weaknesses [39].

Growth mindset learning and feedback literacy were staple inclusions in the multiple regional and national workshops described above. In July 2023, we condensed the material and, with the assistance of a learning technologist, launched a three hour interactive online module. This module incorporates our messages on growth mindset learning and WBAs and teaches, through simulated examples, how to enter learning records, how to review them and how data will be managed in the continuing assessment of the trainee. This online module was made mandatory for all trainees commencing in July 2023, and is also recommended for faculty and trainers.

## Outcomes of Innovation

**Figure 4:**
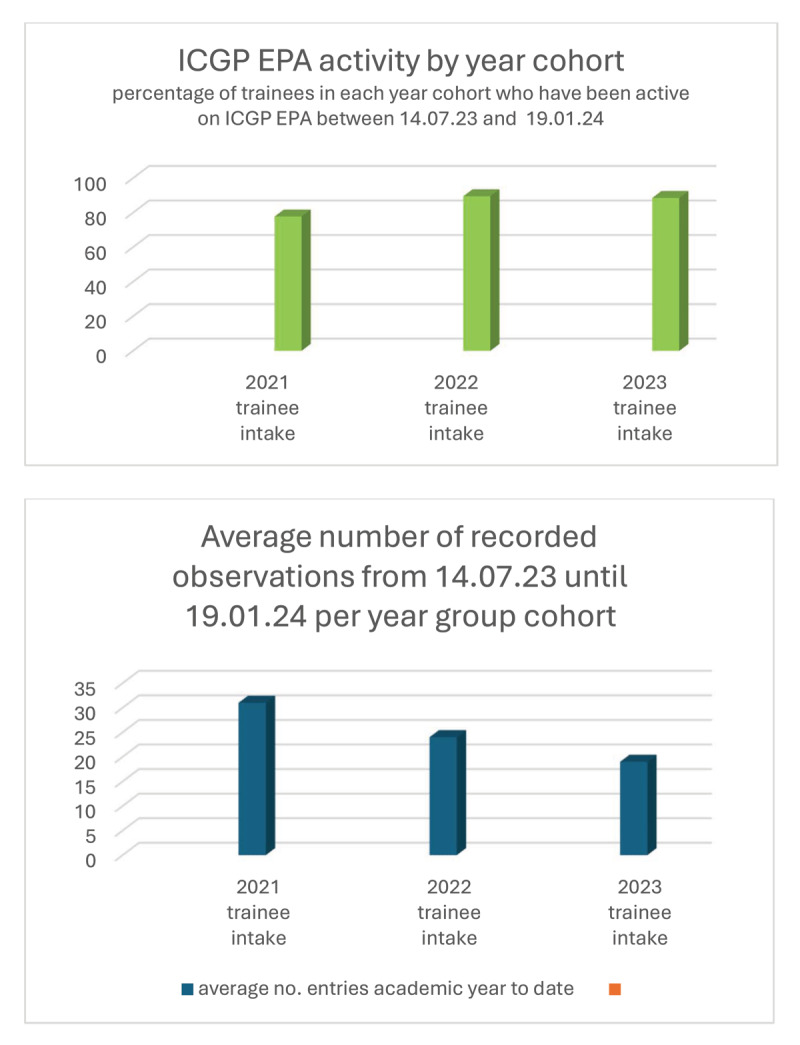
ICGP EPA uptake 6 months post commencement. Uptake of ICGP EPA in first six months of full implementation. This figure demonstrates activity in the form of percentage of trainees in each training year (intake 2021, 2022 and 2023) who are actively using the software, and in the lower graph the average number of entries per year.

The first test of any WBA is its acceptability [14]. Figure 4 shows the trainee uptake of WBA and the average number of recorded entries for each year, six months after commencement. Allowing for trainees on prolonged leave, uptake is almost universal, as might be expected for mandatory WBAs. This uptake was achieved soon after implementation, coming from a position where most trainees previously recorded no WBAs. The average number of entries in the first six months – 30 for third years, 22 for second years and 19 for first years – indicates that trainees are happy to engage with this system; indeed some trainees have entered over 130 entries in six months. The average number of entries is still lower than will be necessary for the competency committees. We believe with time, as trainees and clinical supervisors adapt to the system, and with continued oversight by educational supervisors and EPA champions on each training site, average numbers will increase.

The current standard for assessment validation [40] in medical education is from five sources of validity data: content validity, response process, internal structure, relationship to other variables and consequences. Content validity, a primary focus of validation in EPAs [41], has been established at commencement. In addition to creation of the EPAs through extensive consultation, 10 sample EPAs have been evaluated using the EQUAL validation tool [20] by a panel of eight international experts. Taylor *et al* have recommended 4.07 as the cut-off average score for an EPA to meet the threshold for quality [20]. 9 of the 10 EPAs exceeded this score, the EPA “Advocate for patients” was close with a score of 4.05. Response process data has been gathered in the pilot implementations documenting the engagement with ICGP EPA. More response process data will be gathered in continuing evaluation. Internal structure has been validated for the tools [19] and the dashboard [42]. Data on relations with other variables will be demonstrable by the supervision level progression, compared to exam results and clinical site feedback as trainees advance through their training. Consequences evidence will include examination of the effect of WBA progression data on the number of trainees who are required to undergo additional training. The new system can be defended in relation to patient safety. Use of an entrustment scale enhances reliability [14], there are also checks within the system to establish the veracity of a feedback giver and the truthfulness of recorded narrative feedback. Credibility of the entries logged by the trainee is a review responsibility of the educational supervisor and the competency committee.

One significant setback has been encountered as we have scaled up to include over a thousand trainees. Our original software providers, while responsive to the design and data display requirements of development, were challenged by the requirement to resolve technological glitches experienced by multiple trainees, supervisors and Programme staff, across multiple devices. During the first year of full implementation, continuing IT difficulties led to the Programme changing software providers. The creation of a similar data entry form and migration of all the data from the previous software provider has gone smoothly. However we have lost some functionality in the dashboard display: the single 18 EPA wheel has been replaced by 18 individual pie charts, one per EPA. We hope to improve the functionality of the new version EPA dashboard in the near future through continuing development.

Initial feedback from the training community has been mixed. Some trainees have commented that the platform is “clunky” and “too wordy”, but other trainees embrace the system enthusiastically. Some clinical supervisors have compared the system to sports fitness applications, which motivate as they document physical training. This suggests understanding of the intentional integration, (a feature of PA), of records which have the dual purpose of supporting learning and contributing towards assessment [28]. Educational supervisors like the concepts, although some have stated that interpreting the data can be difficult. A particular strength appreciated by educational supervisors is the ability to rapidly identify outlier trainees on either end of the spectrum.

Competency progression committees are working well in all the training sites using the EPA dashboards and drill down functions to make high stakes decisions. There are multiple observations from multiple observers, mitigating against sampling error, the commonest source of bias [43]. We do not yet have a report on the number of feedback givers per trainee, but this data is logged, and such a reporting function is possible.

Progress towards PA has continued in other elements of the programme. Work has commenced on rearranging the applicable curriculum chapters in line with the 18 EPAs. We do recommend simultaneous transformation of curriculum and assessment where possible. The redeveloped WBA dashboard has been embedded into a new, all-inclusive ePortfolio.

There are many future development, evaluation and research opportunities with our system. Future functionality may include better display of the distribution of the entrustment levels across the domains as well as EPAs, and better interrogation of the data for trainees, trainers and competency committee members. Research opportunities include exploration of the learning culture among our trainees, their perception of how low stakes each entry is, their understanding of feedback and consideration of the effect of feedback on the use of the WBAs.

### Critical Reflection on Process

We attribute the success of our initial implementation to the duration and depth of consultation with the training community, which helped overcome change resistance, and to our continual reference to international developments and literature. In our WBAs, we have shifted the emphasis from numbers to words and images, strengthened the use of feedback, and encouraged mastery-learning. Placing the learner experience at the centre of continuing evaluation of this system will be vital to embedding this system.

Moving the mind-set around assessment from pass/fail to feedback and learning is a disruption of the paradigm of the organisation, shaking deep beliefs [44]. We have engaged our training community in this effort now for seven years. What we found useful was embedding our engagement in feedback literacy and growth mindset learning training, the avoidance of minimum number of entries and framing of these WBAs as “for-learning”. Also useful has been clarification of the role of the clinical supervisor, as mentor and overseer of multiple low stakes assessments and provider of the ITER. But it is the competency committee members who make the high stakes judgement on trainee progression using the accumulated entrustment decisions, feedback entries, ITERs and other data. Medium term stewardship will encourage more entries and greater specificity of feedback.

A significant amount of learner autonomy is encouraged by this flexible system. There is an inherent tension between learner autonomy and patient safety in medical education, which can be addressed by Vygotsky’s concept of scaffolding [45]. Scaffolding is about providing the support structures to help the learner get to the next level of performance. Through the descriptors of competence per EPA and the descriptors per supervision level for each of the domains, our system provides the clear guidance which is one element of the scaffold [45]. Other elements of the scaffold include the trainee seeking feedback, learning how to ask for help [46] and provision by the teacher of constructive, specific and timely feedback [45]. The same features which have been shown to be at the basis of an entrustment decision in an EPA (competency, reliability, humility, integrity and agency) [47] are also features which promote trust in learner autonomy [46].

For our WBAs we considered the validity and reliability of the system as a whole rather than of the tools. St-Onge et al [48] describes three understandings of validity in medical education; as a test characteristic i.e. an intrinsic property of a tool, as an argument based evidentiary chain, i.e. validity belongs to the process rather than to the tool itself, or as a social imperative, i.e. learning for the student and safe qualifying practitioners for society. It is this third understanding which they argue is more aligned with programmatic assessment. For WBAs in particular, Prentice argues that user-tool-context interactions largely determine their validity and reliability [14], in contrast to psychometric testing of the tool itself. The exam components which our trainees undergo are formally validated tools, but the WBAs, in contrast, are in the messy world of day-to-day clinical practice. It is the mosaic of all the data points in the overall portfolio, containing a mixture of tool validated and process validated information which contributes to the high stakes decision of the competency committee.

Whether the duality of purpose; assessment and learning support, of the WBA records is truly achievable has been questioned [49]. Our experience suggests this duality is achievable, through avoidance of summative signals, expectation of multiple entries, and a strong longitudinal educational alliance between teacher and learner [28]. As numbers are strong summative signals, these were avoided where possible, with no numbers attached to entry or display of entrustment scales, no minimum numbers of completion and with visual patterns dominating the accumulated data display of WBAs. Expectation of multiple weekly entries demotes each entry to a quotidian event, which we expect to lessen the tendency to showcase. A strong longitudinal educational alliance has long been a feature of Irish GP training.

This system will grow and evolve, as all curriculum and assessment systems must. While patient safety will remain the prime consideration, the encouragement of mastery learning through intentional design of assessment-for-learning is appropriate in training modern doctors.

## Additional Files

The additional files for this article can be found as follows:

10.5334/pme.1428.s1 Supplementary File 1. EPAs, their domains and descriptors used for GP training in Ireland.

10.5334/pme.1428.s2 Supplementary File 2. Criteria for assessment of WBAs towards high stakes decisions by competency committees.

10.5334/pme.1428.s3 Supplementary File 3. Screenshots of entering and displaying data on our software.
