# Supplementary material for: How We Built Workplace Based Assessment-for-Learning in Irish GP Training
Source: Perspect Med Educ. 2025 Jul 22;14(1):411–22. doi: 10.5334/pme.1428 (PMC12292051; doi:10.5334/pme.1428)
Supplement: Supplementary File 1. — EPAs, their domains and descriptors used for GP training in Ireland. [file pme-14-1-1428-s1.pdf]

Supplementary File 1; EPAs, their domains and descriptors used for GP training in Ireland

List of entrustable professional activities for GP Training in Ireland

EPA 1 Manage the care of children with acute presentations

EPA 2 Manage the care of children with chronic conditions

EPA 3 Manage the health promotion and preventive health care of children

EPA 4 Manage the mental health needs of children

EPA 5 Manage the care of adolescents (12-18) with acute presentations

EPA 6 Manage the care of adolescents (12-18) with chronic conditions

EPA 7 Manage the mental health needs of adolescents (12-18)

EPA 8 Manage the health promotion and preventive health needs of adolescents (12-18)

EPA 9 Manage the care of adults, including older persons, with acute presentations

EPA 10 Manage the care of adults, including older persons, with chronic conditions and complex multimorbidity

EPA 11 Manage the health promotion and preventive health care needs of adults, including older persons

EPA 12 Manage the mental health needs of adults and older persons.

EPA 13 Advocate for patients

EPA 14 Manage a pregnancy

EPA 15 Care of the patient with life limiting illness, palliative care and end of life care needs

EPA 16 Manage investigations

EPA 17 Manage the integration of evidence based therapeutics into patient care and prescribe and manage medications

EPA 18 Manage the GP practice and available resources.

Example of descriptors, mapping and sub-competencies of EPA1, with example of benchmarking.

**EPA 1**	**Manage the care of children with acute presentations**
**Description of the activity**<br>At the end of training the GP is able to assess, manage, triage/prioritise urgent cases, treat and appropriately follow-up the care of the acutely unwell child within the daytime practice and out-of-hours setting. The GP is able to communicate appropriately with the parent and child while maintaining a good doctor-patient relationship in an efficient manner. The GP has a knowledge and understanding of the legal framework pertaining to children and is able to effectively navigate the complexities of family dynamics, recognising and supporting the critical role of	

parents.		
**Example presentations**	Acute respiratory illness, severe gastrointestinal illness, dehydration	
**Medical Council Domain(s) of Good Professional Practice**	• Patient safety and quality of care<br>• Relating to patients<br>• Communication and interpersonal skills<br>• Collaboration and teamwork	• Management (including self-management)<br>• Scholarship<br>• Professionalism<br>• Clinical skills
**Medical Ccouncil Pillar(s) of Professionalism**	Practice; Partnership; Performance	
**Expected proficiency at end of GP Training**	Level 4: Independent practice	

Competencies mapped to the six core domains of the WONCA definition of General Practice.

By the end of training the GP is able to:

1. Primary Care Management	• Recognise acute conditions that can be managed safely within the practice<br>• Recognise the acute conditions requiring monitoring and further review<br>• Make effective use of practice resources, personnel, and systems
2. Person-Centred Care	• Manage the fears and anxieties of parents with an acutely unwell child<br>• Tailor communication to the needs of the parent and child in the context of the history and management plan
3. Specific Problem-Solving Skills	• Prioritise the need for urgent treatment and onward referral<br>• Demonstrate a tailored approach to physical examination of the child<br>• Use a clinical evidence base to inform assessment, diagnosis and treatment/management decisions<br>• Demonstrate safe prescribing in paediatrics<br>• Recognise and respond to child protection issues<br>• Choose, perform and interpret relevant procedures
4. Comprehensive Approach	• Able to address multiple issues and prioritise.
5. Community Orientation	• Use the consultation to approach health promotion and management options and decisions where appropriate<br>Use available local resources effectively.
6. Holistic Approach	• Recognise the complexities of family dynamics<br>• Recognise the implications of cultural context on the consultation

Example of bench marking of the domain of “Specific problem solving skills” in EPA 1

Close supervision	Moderate supervision	Low supervision	Ready for independent practice	Expert, could teach others
Generates a differential diagnosis for presentations<br><br>Aware of child protection issues.	Prioritises the differential diagnosis for presentations<br><br>Understands child protection issues.	Uses an understanding of probability based on prevalence, incidence and natural history to aid decision making<br><br>Habitually considers child protection issues	Use a clinical evidence base to inform assessment, diagnosis and treatment/management decisions<br><br>Demonstrates safe prescribing in paediatrics<br><br>Recognises and responds to child protection issues	Is able to use and justify discretionary judgement in situations of uncertainty or complexity
