# Supplementary material for: How We Built Workplace Based Assessment-for-Learning in Irish GP Training
Source: Perspect Med Educ. 2025 Jul 22;14(1):411–22. doi: 10.5334/pme.1428 (PMC12292051; doi:10.5334/pme.1428)
Supplement: Supplementary File 2. — Criteria for assessment of WBAs towards high stakes decisions by competency committees. [file pme-14-1-1428-s2.pdf]

Supplementary file 2: Criteria for assessment of WBAs towards high stakes decisions by competency committees.

1. Sufficient ICGP EPA data is being logged by the trainee.
2. ICGP EPA records demonstrate attention to their learning by trainees.*
3. The entrustment levels are credible when compared to EPA and supervision level descriptors for the training level.
4. Over time, there is evidence of progression of entrustment levels.
5. Mandatory procedures which require evidence of performance at the level of being ready for independent practice have successfully been achieved.
6. Prior to qualifying from GP training there are some ICGP EPA records for every EPA that shows the trainee has reached the level of entrustment of being ready for independent practice. The entrustment records across all EPAs and across the entire duration of training should be of a distribution with which the CPC is satisfied.

*This is determined by assessing the distribution of entries over the EPAs and the recorded narrative feedback is of good quality with evidence of rich and specific feedback.
