# Supplementary material for: How We Built Workplace Based Assessment-for-Learning in Irish GP Training
Source: Perspect Med Educ. 2025 Jul 22;14(1):411–22. doi: 10.5334/pme.1428 (PMC12292051; doi:10.5334/pme.1428)

Supplementary File 3; Screenshots of entering and displaying data on our software.

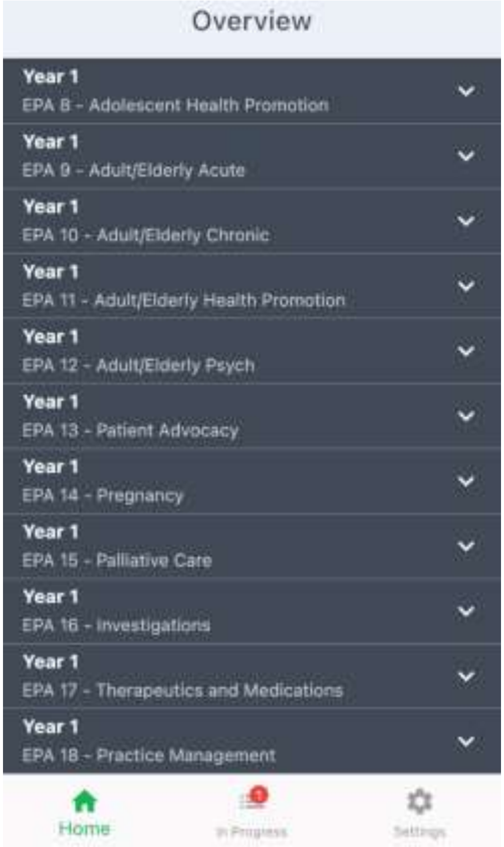

This image shows the first screen on opening up the software. Here the trainee chooses an EPA appropriate to the clinical activity on which they seek feedback

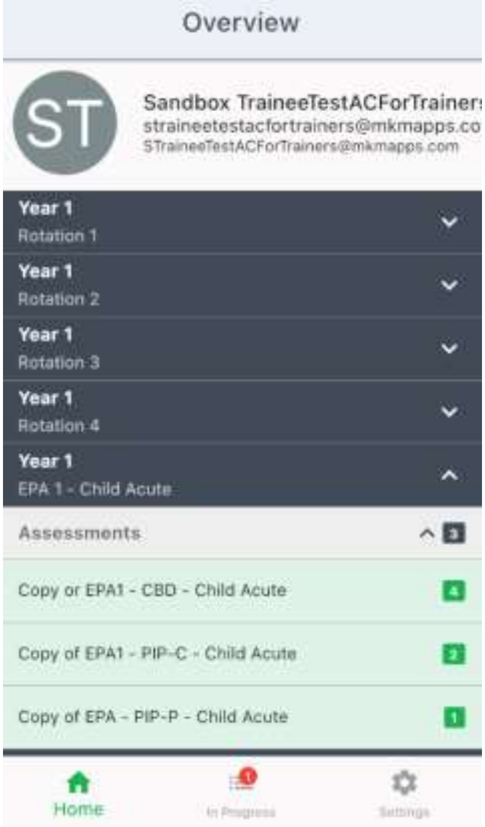

Having chosen the EPA 'acute presentation in a child', clicking on this reveals the three tools which the trainee can [illegible] cussion when the feedback is based on a conversation without the supervisor directly observing their consultation.

They use a PIP-C, performance in practice – consultation, where the part of the consultation under discussion has been observed, e.g. history taking.

They use a PIP-P, performance in practice – procedure/physical exam where this has been directly observed, e.g. administration of a nebuliser.

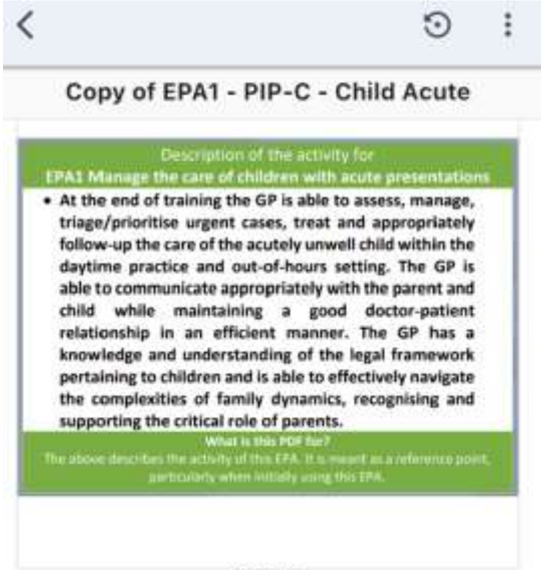

01.PNG

Having chosen a tool in an EPA, the trainee can remind themselves of the descriptor of a competent doctor in that EP

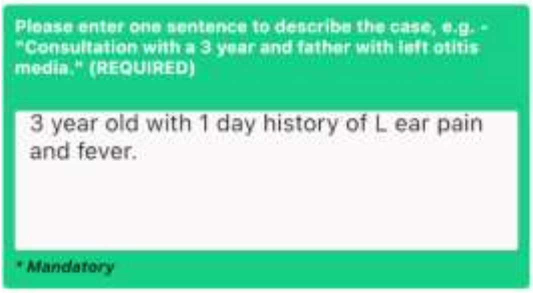

They then enter a short description of the case, ensuring anonymity of the patient.

They then must choose one of the six domains on which to focus their feedback for this clinical activity.

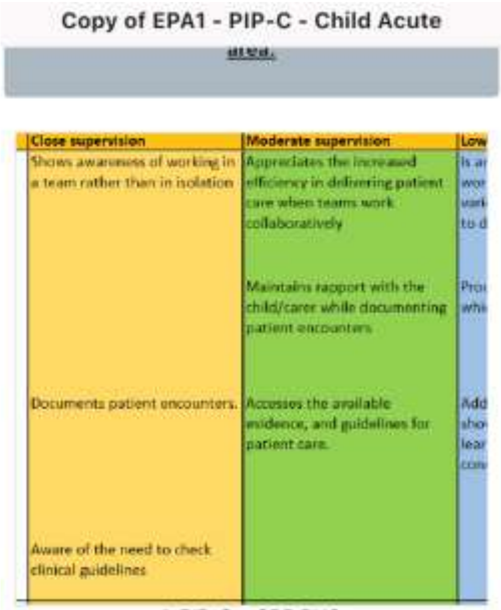

1. PIP-C + CBD.PNG

is example the domain ‘Primary Care Management’ has been chosen.

The descriptors for the levels of supervision are available to both the trainee and the clinical supervisor as they discuss feedback.

It is the top of this screen, just out of view where the statement is situated:

‘competence/supervision levels can vary considerably, therefore the judgements in domains relate to a given case at a given point in time and are not an overall judgement of a trainee’s competence in the area’.

The appropriate level of supervision is chosen from a drop down list which contains all five levels of supervision;

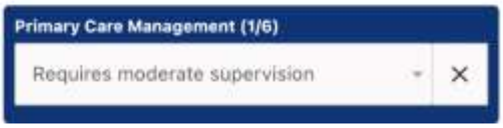

Close supervision

Moderate supervision

Low supervision

Ready for independent practice

Expert

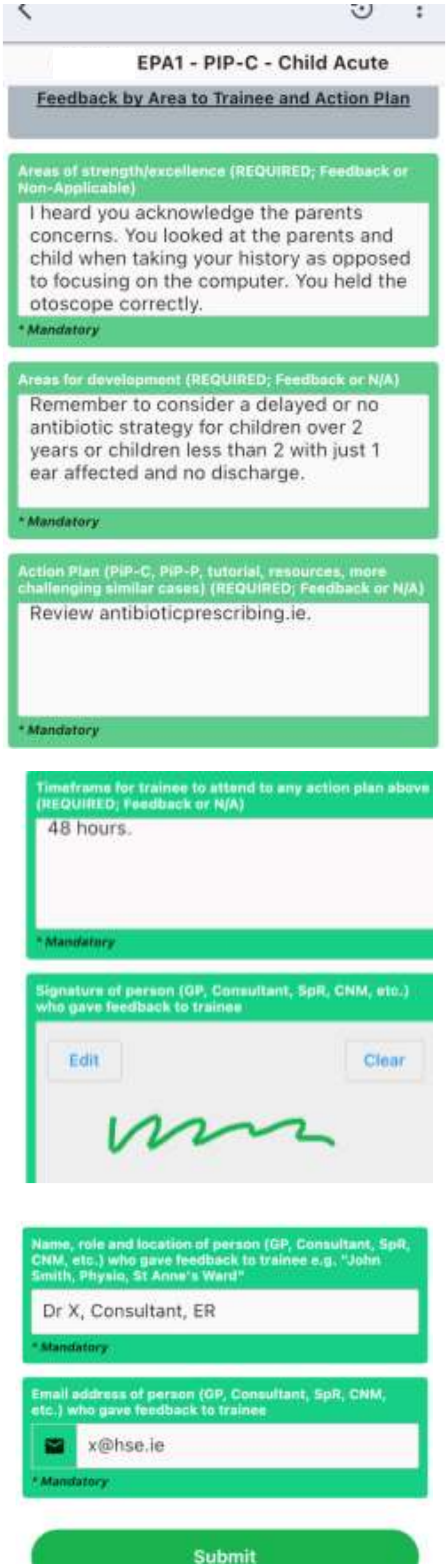

EPA1 - PIP-C - Child Acute

Feedback by Area to Trainee and Action Plan

Areas of strength/excellence (REQUIRED; Feedback or Non-Applicable)

I heard you acknowledge the parents concerns. You looked at the parents and child when taking your history as opposed to focusing on the computer. You held the otoscope correctly.

* Mandatory

Areas for development (REQUIRED; Feedback or N/A)

Remember to consider a delayed or no antibiotic strategy for children over 2 years or children less than 2 with just 1 ear affected and no discharge.

* Mandatory

Action Plan (PiP-C, PiP-P, tutorial, resources, more challenging similar cases) (REQUIRED; Feedback or N/A)

Review antibioticprescribing.ie.

* Mandatory

Timeframe for trainee to attend to any action plan above (REQUIRED; Feedback or N/A)

48 hours.

* Mandatory

Signature of person (GP, Consultant, SpR, CNM, etc.) who gave feedback to trainee

Edit | Clear

Name, role and location of person (GP, Consultant, SpR, CNM, etc.) who gave feedback to trainee e.g. "John Smith, Physio, St Anne's Ward"

Dr X, Consultant, ER

* Mandatory

Email address of person (GP, Consultant, SpR, CNM, etc.) who gave feedback to trainee

x@hse.ie

* Mandatory

Submit

Feedback is recorded, the action plan and the time scale for that action plan are set, and the feedback giver signs the form, identifying themselves.

Display of data

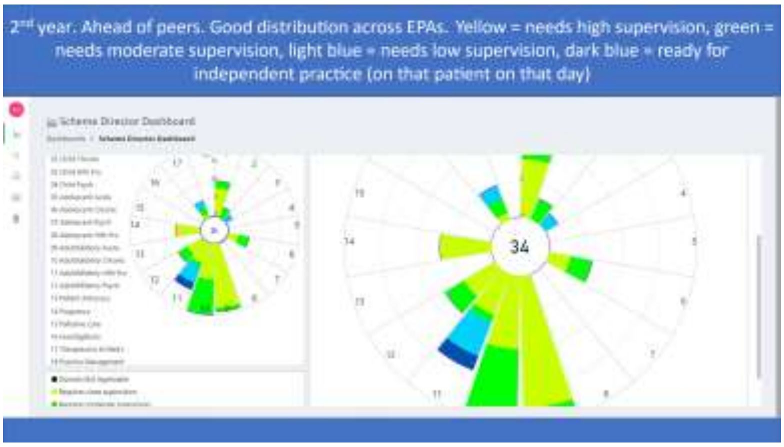

First data display

The big wheel shows the entries per EPA for this trainee for the training year to date. They are colour coded according to supervision level:

Close supervision = yellow

Moderate supervision =light green

Low supervision =dark green

Ready for independent practice - dark blue

Expert = purple

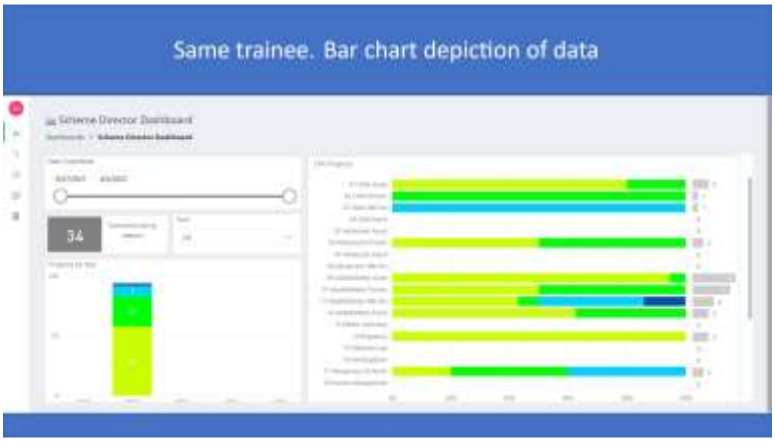

The trainee can also see their data in bar chart format. It is this view which allows drilling through to look at detail.

Drill down can display whole EPAs or those entries associated with a particular supervision level.

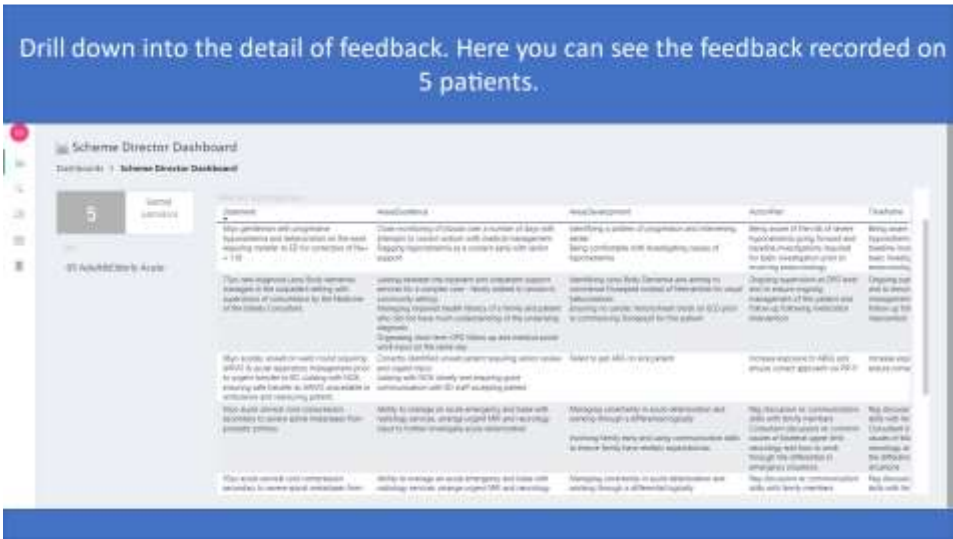

Cohort dashboard:

Here is how a program director can view several trainees dashboards at once. This enables rapid understanding of the general progress of their cohort of trainees, and quick spotting of outliers.

This view is not available to the trainee.

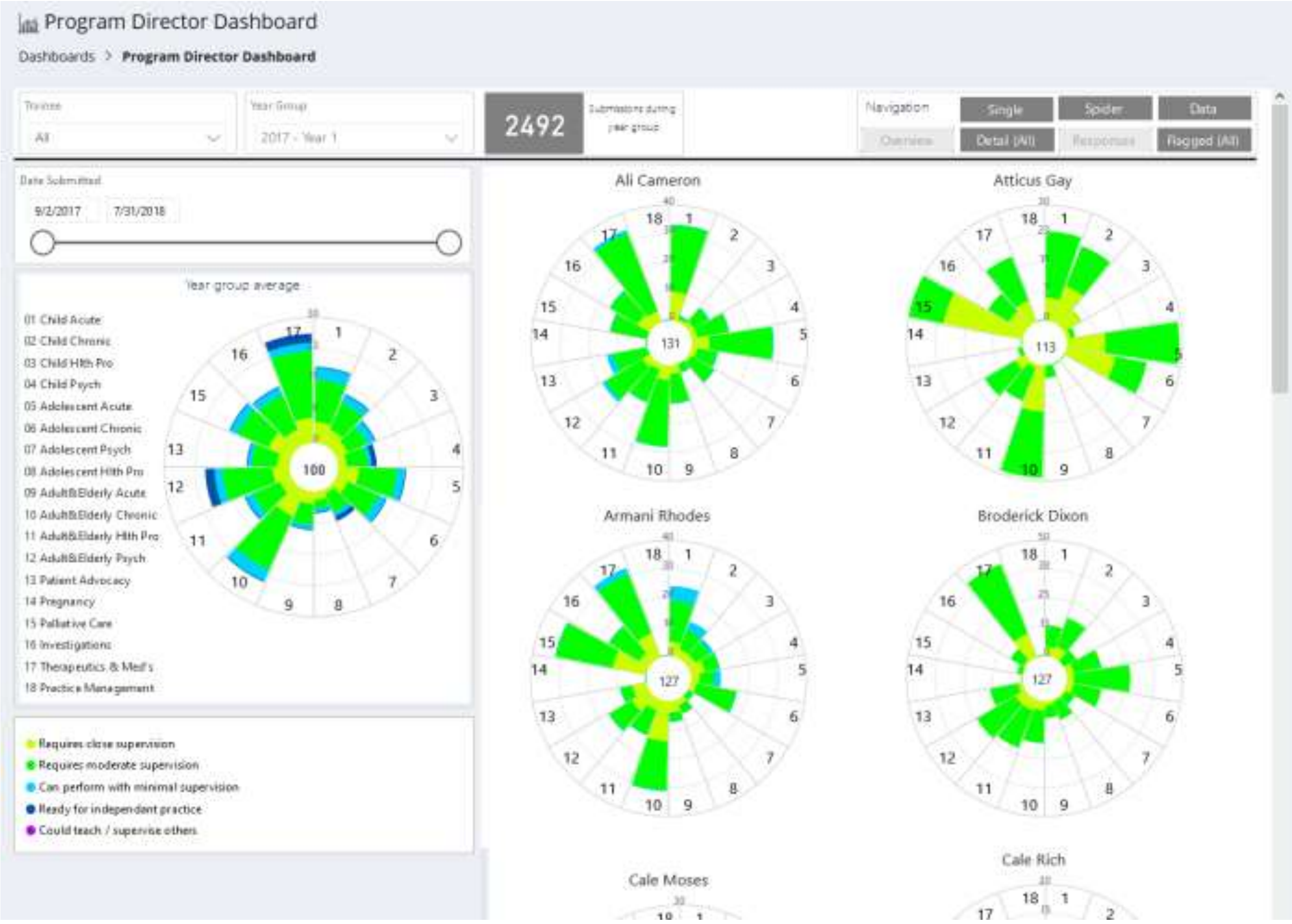

Supplement: Supplementary File 3. — Screenshots of entering and displaying data on our software. [file pme-14-1-1428-s3.pdf]
